# A Case Report of a Coin Stack Masquerading as a Button Battery

**DOI:** 10.5070/53886

**Published:** 2026-07-31

**Authors:** Peyton J Ware, Carter Ware, Amanda H Bjornstad, Heather M Kuntz, Timothy P Young

**Affiliations:** * Loma Linda University School of Medicine, Laboratory for Innovations in Medical Education, Loma Linda, CA; ^ Texas Tech University Health Sciences Center El Paso, Department of Radiology, El Paso, TX; † Loma Linda University Medical Center, Department of Emergency Medicine, Loma Linda, CA

## Abstract

Children presenting to emergency departments who are found to have flat, round esophageal foreign bodies (FB) are often transferred to sites capable of foreign body removal. Coins are the most common FBs ingested in pediatric patients. However, button battery (BB) ingestions are a high-risk esophageal FB that can lead to severe injury. It is imperative to distinguish between these two radiographically similar objects to ensure expedited removal of BBs. The halo sign and step-off sign are characteristic of BBs. The presented case demonstrates an exception, where a coin stack (CS) mimicked a BB. A six-year-old female arrived at a tertiary care center as a transfer from an outside hospital with concern for BB ingestion. Her presenting symptoms were vomiting, gagging, and drooling. Her chest x-ray showed a radio-opaque, esophageal FB with halo and step-off signs. The circular FB was urgently removed and proved to be a nickel overlying a quarter. Due to the relative rarity of CSs and the difficulty to radiologically distinguish from high-risk BBs, CSs should be treated as BB ingestions—the FB should be removed urgently, and the final object identification should be determined operatively.

**Topics:**

Button battery, halo sign, step-off sign, coin stack, esophageal foreign body.

**Figure:**
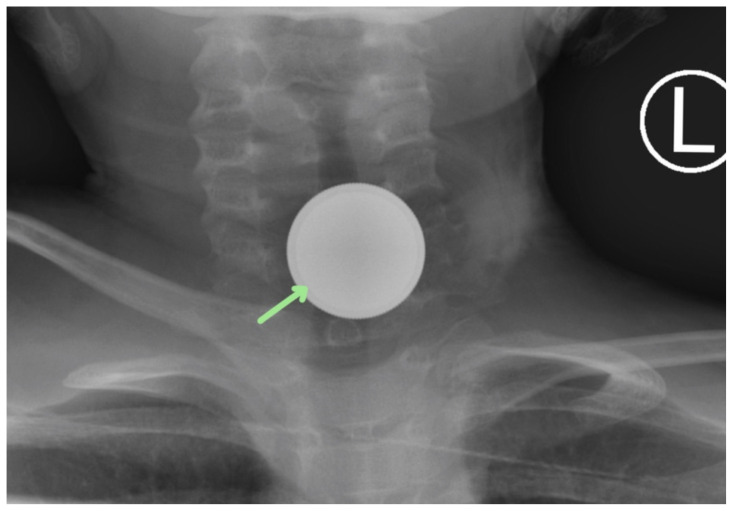


**Figure:**
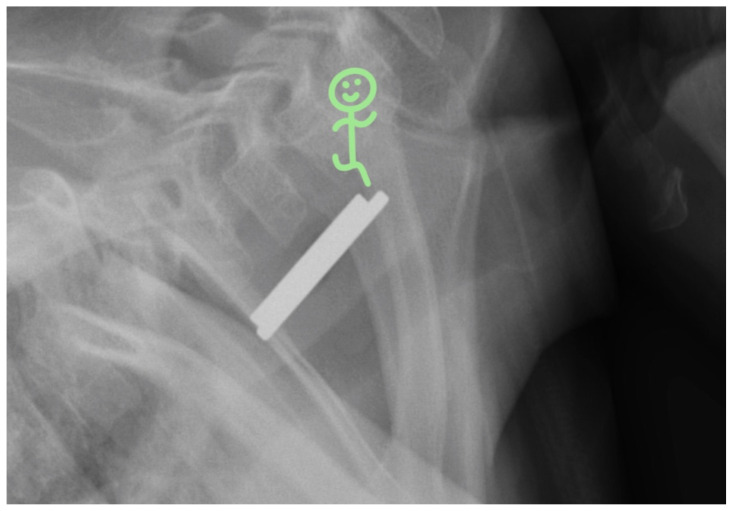


## Brief introduction

A young child with an esophageal foreign body can present with dysphagia, hypersalivation, gagging, or vomiting after a choking episode.^1^ Coins are the most commonly ingested foreign body (FB) in the pediatric population.^2^ Button batteries (BBs) are less common, high-risk FBs that can cause devastating esophageal injury.^3^ BBs cause injury from an electrolysis reaction that results in coagulative necrosis at the cathode and liquefactive necrosis at the anode.^4^ While both BBs and coins are round, metallic FBs, two key radiographic signs distinguish a BB from a coin: the halo sign and the step-off sign.^5^,^6^ Though all FB ingestions carry potential for complications, the speed and severity at which BBs cause injury necessitate early identification to ensure prompt treatment.^4^,^8^ In this report, we present the case of a child who ingested a stack of coins that mimicked a BB.

## Presenting concerns and clinical findings

A six-year-old female with autism presented to an outside hospital (OSH) emergency department (ED) with vomiting, gagging, and drooling after reportedly swallowing coins. The OSH abdominal x-ray (XR) was unremarkable. The single-view OSH chest x-ray (CXR) identified a round FB in the lower cervical esophagus. Anteroposterior (AP) and lateral soft tissue neck XRs showed a FB concerning for a BB: a flat, round metallic foreign object measuring 2.5 cm in diameter with a halo sign and stepped edges. The patient was transferred to the closest children’s ED for further management and foreign body removal.

Upon arrival, the patient was well-appearing with intermittent gagging and vomiting episodes. Her temperature was 98.6°F, heart rate was 135 beats per minute, respiratory rate was 22 breaths per minute, blood pressure was 84/55, and her oxygen saturation was 98% on room air. She had no visible oropharyngeal FB or edema, stridor, unilateral wheezing, chest wall crepitus, or abdominal tenderness to palpation. Additionally, the patient said “money,” which corroborated the mother’s report that the patient swallowed coins.

The step off sign on the lateral neck XR was noted to be asymmetric. While the asymmetry was unexpected for a BB, the otolaryngology (ENT) team was urgently consulted due to the possibility of a high-risk BB ingestion. The patient’s mother told the ENT team that her daughter had been playing with money, and BBs were not available in their home. Repeat AP and lateral CXRs were similar to OSH images and showed the object had not moved from the proximal esophagus.

## Significant findings

The two images provided are OSH AP and lateral XRs of the patient’s neck. The OSH AP view demonstrates a halo sign (green arrow), while the lateral view demonstrates a step-off sign (stick figure). These are key findings with BB ingestions. The halo sign is the presence of two concentric rings on an anterior view XR.^5^,^6^ Although all United States coins have a subtle ridge that can be mistaken for double concentric rings, the halo sign of BBs and coin stacks (CSs) is much more well defined.^9^ Additionally, quarters and dimes have ruffled edges on anterior-posterior XRs. The step-off sign is the indentation of FB edges that looks similar to stairs on a lateral CXR. In this case, note the asymmetric difference in step lengths on the lateral neck XR view.

## Patient course

The consulting ENT team agreed that the XRs showed a halo and asymmetric step-off sign. The on-call radiologist expressed concern for a possible BB ingestion, given the stepped edge of the esophageal FB. The patient was urgently taken to the operating room where it was determined that the foreign body was a CS: a quarter and nickel adhered to one another by saliva were removed from the esophagus. The patient was monitored for two hours in the post-anesthesia care unit and was discharged home.

## Discussion

This case highlights an interesting instance of a CS presenting with radiographic findings similar to those of a BB. The coins were removed emergently for a definitive diagnosis and to avoid the complications known to occur with BBs. Other similar cases of a CS mimicking a BB have been reported.^7^,^10^–^14^ Conversely, up to 10% of BBs are initially misdiagnosed as coins on XR.^3^ In these cases, the potential consequences can be devastating. The halo and step-off signs are intended to distinguish a BB from a single coin. CSs and BBs are more difficult to differentiate due to their shared halo and step-off signs on radiographs, produced by their similar size, shape, and contours. Artificial intelligence (AI) can effectively differentiate a single coin and BB, but multiple coins remain relatively indistinguishable from a BB to an AI algorithm.^14^ CSs are also quite rare. In one study of 139 pediatric foreign body ingestions, 91.4% swallowed one coin, 5.8% swallowed multiple coins, and 3.6 % swallowed a button battery.^13^ Of the eight children who swallowed multiple coins, only one presented as a CS. Of six XRs concerning for a BB ingestion, only one was a CS. As in our case, this one CS presented with radiographs showing a halo and step-off sign.^13^ In light of the rarity of CSs, the difficulty with which they are distinguished from BBs, and given the high-risk nature of BB ingestions, treatment of CSs that mimic BBs is the same as true BB ingestions: remove the esophageal FB without delay.

While the majority of pediatric esophageal foreign bodies occur in toddlers, children with neurodevelopmental delay may present with a foreign body ingestion at an older age.^2^,^15^ An Italian study of 457 children with foreign body ingestion included a subgroup of 16 patients with autism who presented at a mean age of 9.8 years.^15^ This study and our case demonstrate the importance of keeping foreign body ingestion in the differential diagnosis for an older child with limited verbal capacity.

It is important to note that, despite the mother’s certain report of immediate access to coins with no household BBs, the patient was still managed with the worst-case scenario in mind. Caregiver certainty regarding a child’s FB ingestion has a sensitivity of 86.8% but only 19.8% specificity.^1^ For example, of the five patients with esophageal BB in the previously mentioned study, only two had history concerning for BB ingestion.^13^ Lack of caregiver suspicion of a BB ingestion can lead to prolonged BB impaction.^3^ History that misdirects clinical management can delay BB removal and lead to esophageal perforation, fistulas, strictures, and other injuries.^3^, ^16^ Even when a single coin is suspected, Frumkin and Lanker urge clinicians to “look again” at images of esophageal FB to avoid “the potential dangers of confirmation bias.” If radiograph findings are concerning for BB ingestion, care should not be delayed due to an inconsistent history because a delay in care risks detrimental injury from a potential BB ingestion.^17^

While this case highlights an interesting phenomenon where a stack of coins mimicked a BB, the degree of difficulty with which the two FBs are distinguished radiographically and the potential severity of injury caused by a BB dictate that the final determination be made operatively.

## Supplementary Information








